# Differential Performance of Distribution Shifts Between Endangered Coniferous and Broad-Leaved Tree Species in Subtropical China Under Climate Change

**DOI:** 10.3390/plants15030515

**Published:** 2026-02-06

**Authors:** Jie Miao, Yan Xu, David Kay Ferguson, Yong Yang

**Affiliations:** 1 Co-Innovation Center for Sustainable Forestry in Southern China, College of Life Sciences, Nanjing Forestry University, Nanjing 210037, China; miaoj@njfu.edu.cn (J.M.); xuy@njfu.edu.cn (Y.X.); 2 Department of Paleontology, University of Vienna, 1090 Vienna, Austria; david.kay.ferguson@univie.ac.at

**Keywords:** climate change, species distribution models, MaxEnt, rare and endangered tree species, subtropical evergreen broad-leaved forests

## Abstract

Global warming has become one of the most serious threats to biodiversity. However, the responses of endangered tree species in subtropical regions to climate change and their potential distribution shifts remain elusive. In this study, we selected nine rare and endangered tree species in the subtropical forests of China encompassing both coniferous and broad-leaved groups, and conducted an assessment of their suitable distribution patterns and spatial shifts under current and future climate scenarios (SSP126, SSP370, and SSP585). For this we utilized an optimized MaxEnt model integrating multidimensional environmental variables including climate, soil, and topography. The results show that the model has high predictive accuracy after parameter optimization, with mean AUC values exceeding 0.98 for both broad-leaved and coniferous tree species. Our analysis of environmental factors indicates clear differences in distribution-limiting factors between the two functional groups. Broad-leaved species are primarily constrained by temperature-related variables, particularly the mean temperature of the coldest quarter (Bio11) and the mean diurnal range (Bio2), whereas coniferous species are more sensitive to moisture conditions, with the precipitation of the driest quarter (Bio17) as the key limiting factor for their potential distributions. Under current climatic conditions, highly suitable habitats for both functional groups are mainly concentrated in the middle and lower reaches of the Yangtze River. Under future climate scenarios, broad-leaved species are in general expected to expand in marginal areas, while coniferous species show pronounced scenario dependence, with significant contractions occurring under certain scenarios and time periods. Despite the evident changes at distribution margins, the overall shifts in the centroids of potential distributions for both functional groups will be limited, with core suitable areas remaining relatively stable. This study reveals differences in the spatial response patterns between conifers and broad-leaved trees, and provides a scientific basis for the development of differentiated conservation strategies and the identification of conservation priority areas under climate change.

## 1. Introduction

Under global warming, continuous increases in temperature accompanied by pronounced changes in precipitation regimes have become key factors driving transformations in the structure and function of terrestrial ecosystems. Since the 20th century, the global mean temperature of land and oceans has risen by approximately 1 °C and is projected to increase further within this century [1,2]. Climate change is manifested not only as a long-term warming trend, but also as a factor transforming the spatial and temporal distribution of precipitation, increasing the frequency of extreme heat and drought events, and causing a sustained rise in atmospheric evaporative demand; collectively, these changes have a profound influence on plant growth, reproduction, and resource utilization processes [3,4]. Previous studies have indicated that climate change is reshaping plant geographic distribution patterns by modifying species’ physiological tolerance thresholds, phenological rhythms, and water balance conditions, with especially pronounced effects on endemic plants [5,6]. In this process, species track suitable climatic conditions through range shifts; however, when the rate of climate change exceeds their dispersal or adaptive capacity, they face risks of potential habitat contraction, population decline, or even local extinction [7,8]. Therefore, assessing the changes in potential plant distribution patterns under global warming is of critical importance for understanding the response mechanisms and for formulating effective conservation strategies.

Subtropical regions are particularly sensitive to climate change, with marked spatial heterogeneity in vegetation responses to variations in topography, temperature, and moisture [9]. As one of the global biodiversity hotspots, the subtropical evergreen broad-leaved forests of China are not only home to a rich mixture of plant taxa, but also harbor numerous endemic and endangered tree species with restricted distributions and small population sizes [10]. Previous studies have indicated that, under continued warming, the potentially suitable habitats of tree species in this region may undergo substantial reorganization, with some species experiencing range expansion at the margins, while others will face the dual pressures of habitat fragmentation and functional degradation [2,11,12]. However, the potential distributional responses of endangered tree species to global warming remain poorly understood, particularly with regard to the differences between conifers and broad-leaved species.

Many endemic and endangered tree species are found in the subtropical evergreen broad-leaved forests of China. These species not only possess unique evolutionary status, but have important ecological and conservation value at both global and regional scales [13,14]. We selected a set of representative species based on their key ecological functions within forest communities. For example, phylogenetically distinctive paleoendemic taxa such as *Shaniodendron subaequale* play an irreplaceable role in maintaining ecosystem genetic diversity and functional diversity [15]. Although these rare tree species generally do not dominate the entire forest or constitute the main biomass, they often act as critical structural components in specific microhabitats or function as locally dominant species. For instance, *Pseudotsuga gaussenii* and *Pseudolarix amabilis* contribute to enhancing the structural stability and vertical complexity of mixed conifer–broad-leaved forests [16,17], whereas taxa such as *Ulmus elongata* and *Litsea auriculata* provide important ecological resources for regional pollinator and seed dispersal networks [18,19]. In addition, many of these species are remnants of ancient lineages (paleoendemics), generally characterized by narrow distribution ranges and high sensitivity to climatic and habitat conditions [15]. Under natural conditions, these species commonly exhibit mixed growth patterns, coexisting with widespread subtropical tree species and forming forest communities with high structural diversity and strong resilience [14]. They also possess marked differences in functional types, niche requirements, and responses to environmental change. This makes them suitable for studying the distributional characteristics and potential response patterns in subtropical evergreen broad-leaved forests [15,19,20].

Species distribution models (SDMs) provide important tools for quantitatively assessing the relationships between environmental factors and species distributions and have been widely used to predict potential distribution ranges under climate change scenarios, identify climate refugia, and support conservation decision-making [21,22]. Of the various SDM approaches, such as the generalized linear model (GLM), generalized boosting model (GBM), and random forest model (RF), the maximum entropy model (MaxEnt) has been widely used in forest ecology and biogeography due to its low sample size requirements, stable predictive performance, and ease of integration with geographic information systems [23,24]. In recent years, with the introduction of parameter optimization methods and multi-source environmental data, the MaxEnt model has been effective in reducing overfitting and enhancing prediction accuracy [12,22,25].

The present study focuses on nine rare and endangered tree species distributed in the subtropical evergreen broad-leaved forests of China. Using an optimized MaxEnt model and integrating multidimensional environmental variables, including climate, soil, and topography, we systematically assessed their potential distribution patterns under current and future climatic scenarios. The objectives of this study were to (1) identify the main environmental drivers shaping the distributions of rare coniferous and broad-leaved tree species; (2) characterize potential suitable habitats under different climate scenarios; and (3) provide scientific evidence for the conservation of rare tree species in subtropical regions under climate change. By conducting an integrated analysis of potential distributions across multiple species and functional groups, this study contributes to a deeper understanding of the stability of subtropical forest ecosystems and the recognition of conservation priority areas.

## 2. Results

### 2.1. Parameter Optimization and Predictive Accuracy Validation

**Figure 1:**
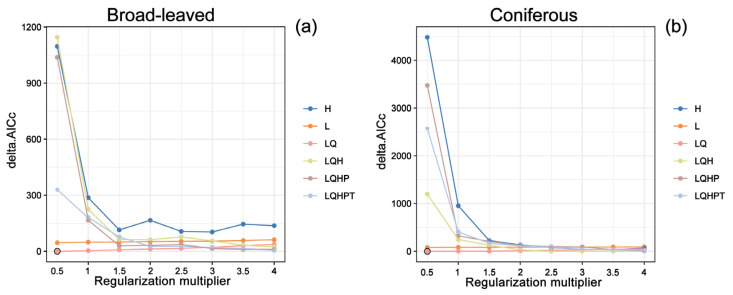
Distribution of delta.AICc values across various combinations of feature classes and regularization multipliers (RMs) in the MaxEnt model. (**a**) broad-leaved species; (**b**) coniferous species.

The configuration of the MaxEnt model was optimized utilizing the ‘ENMeval’ package in R. For both broad-leaved and coniferous species, the combination of a Regularization Multiplier (RM) of 0.5 and Linear and Quadratic (LQ) features achieved the minimum delta.AICc value. This indicates that the selected parameters provided the highest level of model parsimony and effectively mitigated the risk of overfitting according to the Akaike Information Criterion. Consequently, this parameter set was established as the optimal configuration for subsequent simulations (Figure 1a,b).

**Figure 2:**
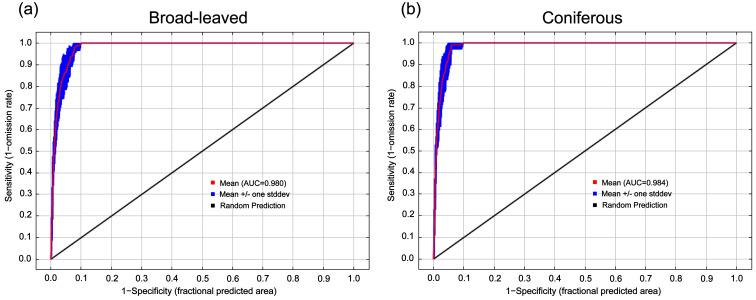
Receiver Operating Characteristic (ROC) curves evaluating the performance of the optimized MaxEnt models. (**a**) broad-leaved species; (**b**) coniferous species.

Based on the Receiver Operating Characteristic (ROC) analysis (Figure 2a,b), the optimized models exhibited exceptional predictive performance. The mean training Area Under the Curve (AUC) values for broad-leaved and coniferous species were 0.980 and 0.984, respectively. These results, which significantly exceed the random prediction threshold of 0.5, demonstrate the high reliability and robust discriminative capacity of the model in identifying the potential habitats of the target species.

### 2.2. Dominant Environmental Factors Impacting the Distribution of Broad-Leaved and Coniferous Taxa

**Figure 3:**
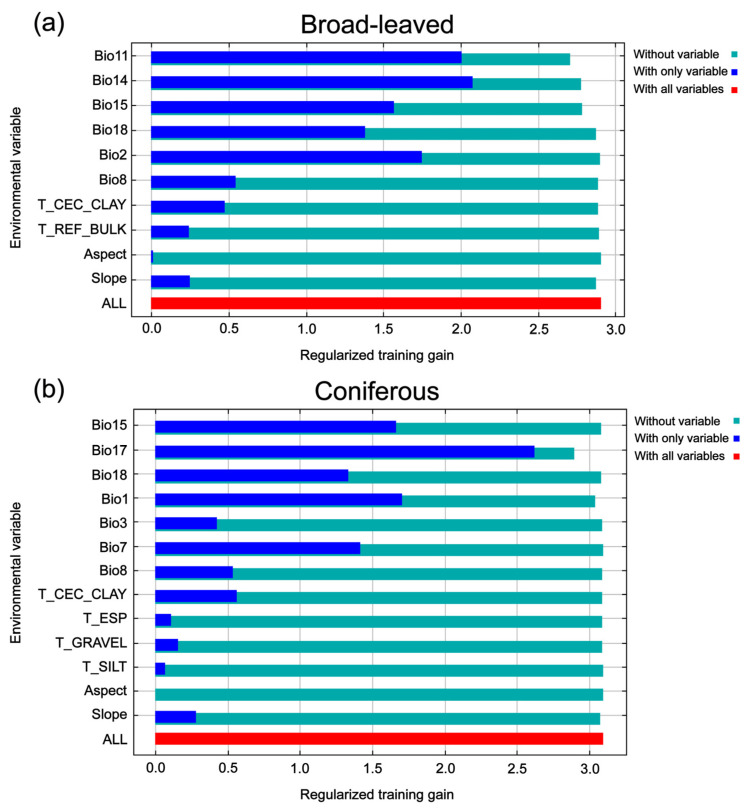
Jackknife test results showing the effects of individual environmental variables on the potential distributions of broad-leaved (**a**) and coniferous (**b**) species.

The analysis of environmental contributions and Jackknife tests revealed that the spatial distribution of the studied species is primarily governed by climatic factors, with clear divergence in ecological requirements between the two functional groups. For the broad-leaved species, the model was predominantly driven by thermal factors. The Jackknife test for this group indicated that while precipitation of the driest month (Bio14) provided the most useful information when used in isolation, Bio11 exerted the greatest influence on the model gain when omitted, suggesting that it contains unique information not captured by other variables (Figure 3a). In contrast, the coniferous species exhibited a higher sensitivity to moisture availability, while the Jackknife test confirmed that Bio17 not only possessed the highest independent explanatory power but also caused the most significant reduction in model gain when excluded, highlighting its indispensable role (Figure 3b). While topographic factors like slope and aspect showed marginal influence in both groups, edaphic properties such as topsoil CEC (clay) (T_CEC_CLAY) provided additional explanatory power.

### 2.3. Potentially Suitable Habitat Distribution Under Current Climatic Conditions

**Figure 4:**
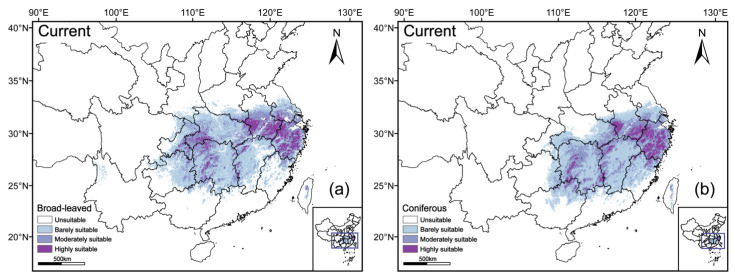
Potentially suitable habitats for broad-leaved (**a**) and coniferous (**b**) tree species under current climatic conditions.

Under the current climate scenario, the MaxEnt model predicted the potentially suitable habitats for rare and endangered broad-leaved and coniferous tree species within the subtropical evergreen broad-leaved forest region of China (Figure 4). Suitable habitats for broad-leaved species are mainly distributed in eastern and central China, and particularly concentrated in the middle and lower reaches of the Yangtze River Basin. Highly suitable areas are primarily located in Zhejiang, southern Jiangsu, and western and southeastern Anhui, but are also sporadically found in parts of Hunan, Hubei, Jiangxi, Chongqing, Guizhou, and Taiwan, forming relatively continuous core regions. Moderately suitable habitats extended outwards from these core areas, whereas barely suitable habitats were more scattered and mainly occurred in Henan, Yunnan, Shaanxi, Sichuan, Fujian, and Guangdong. The spatial distribution pattern of coniferous species was generally consistent with that of broad-leaved species, with highly suitable habitats likewise concentrated in Zhejiang, southern Jiangsu, and western and southeastern Anhui, while moderately and barely suitable habitats are continuously distributed surround the high-suitability centers.

**Figure 5:**
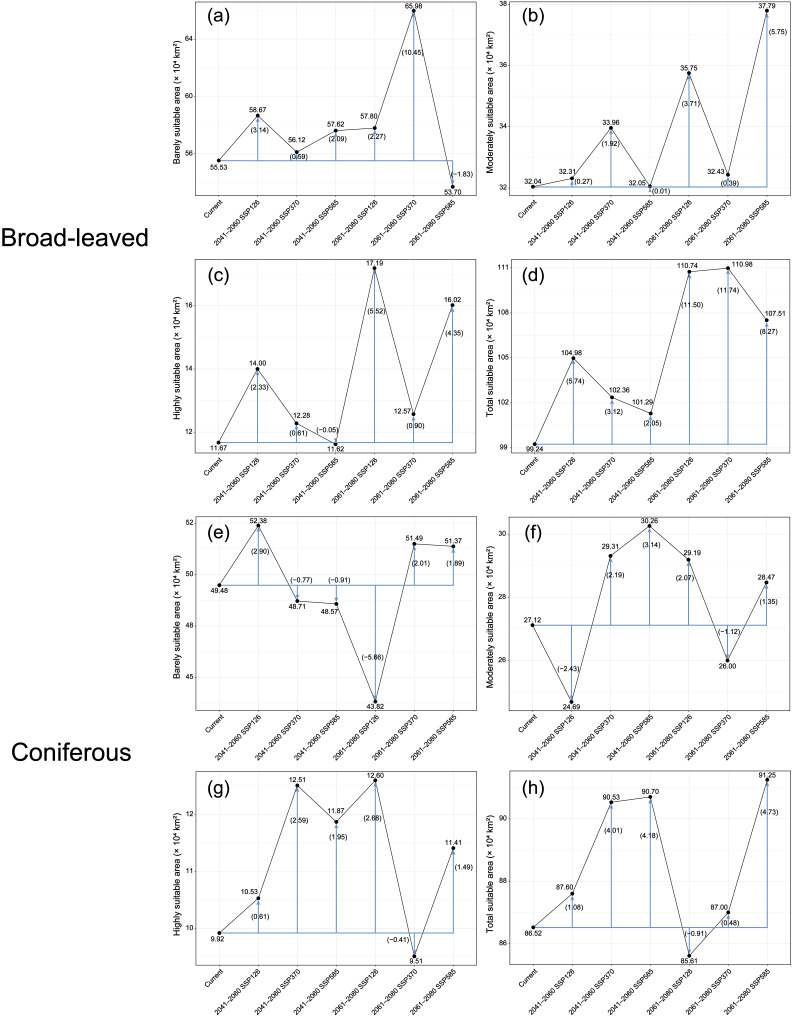
Suitable habitats for broad-leaved and coniferous tree species under current and different future climate scenarios. Panels (**a**–**d**) show the areas of barely suitable, moderately suitable, highly suitable, and total suitable habitats for broad-leaved species, while panels (**e**–**h**) present the corresponding results for the conifers. Black lines represent absolute changes in habitat area across different time periods (×10^4^ km^2^), while blue arrows indicate changes relative to the current period, with values shown in brackets.

Area analysis further indicated significant differences in the extent of suitable habitats between the two plant groups (Figure 5). The total suitable habitat area for broad-leaved species was 99.24 × 10^4^ km^2^, of which barely suitable habitats accounted for the largest proportion (55.53 × 10^4^ km^2^), followed by moderately suitable (32.04 × 10^4^ km^2^) and highly suitable habitats (11.67 × 10^4^ km^2^). In comparison, the total suitable habitat area for coniferous species was smaller (86.52 × 10^4^ km^2^), with barely suitable, moderately suitable, and highly suitable habitats covering 49.48 × 10^4^ km^2^, 27.12 × 10^4^ km^2^, and 9.92 × 10^4^ km^2^, respectively; the proportion of highly suitable habitats was lower and more restricted.

### 2.4. Projected Habitats Under Future Climate Scenarios

**Figure 6:**
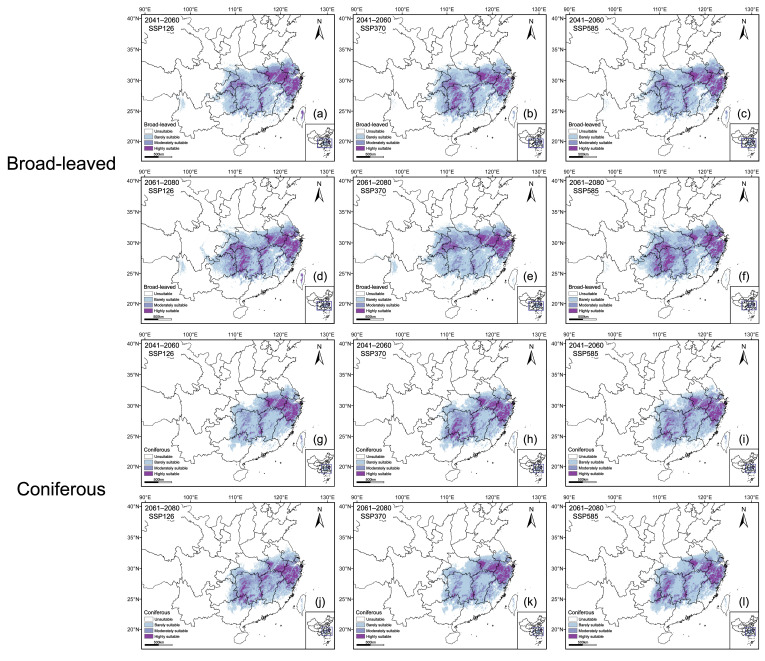
Projected spatial distributions of broad-leaved (**a**–**f**) and coniferous (**g**–**l**) species under various future climate scenarios.

Based on the predictions of the optimized MaxEnt model, the distributions of rare and endangered broad-leaved and coniferous plants are expected to undergo significant changes during 2041–2060 and 2061–2080. This will mainly involve adjustments to the extent of suitable habitats and the proportions of different suitability classes (Figure 5 and Figure 6). For broad-leaved plants, the area under future climatic conditions generally expands compared with the current period, although the magnitude of change depends on the scenario (Figure 5d). Highly suitable habitats encourage expansion with the exception of SSP585 during 2041–2060, and cover relatively large areas under the SSP126 and SSP585 scenarios for 2061–2080 (Figure 5c). In moderately suitable habitats the response is more variable, but on the whole tend to expand relative to the current period (Figure 5b). In contrast, barely suitable habitats increase under most future scenarios, but show a pronounced decline under the SSP585 scenario for 2061–2080 (Figure 5a).

For coniferous plants, the total suitable habitat area exhibits a pattern of ‘initial increase–subsequent decrease–further increase,’ with a slight expansion under all scenarios during 2041–2060, followed by a significant contraction under the SSP126 scenario for 2061–2080 (Figure 5h). The changing trend of highly suitable habitats is generally consistent with that of the total suitable habitat area, but a marked contraction is expected to occur in the SSP370 scenario for 2061–2080 (Figure 5g). Barely suitable habitats show a similar pattern and decline significantly under the SSP126 scenario for 2061–2080 (Figure 5e).

Under all future climate scenarios, suitable habitats for both plant groups tend to be concentrated in eastern and central China, with the core distribution areas largely overlapping those of the present day (Figure 6). Moreover, in highly suitable habitats both plant groups tend to stay put in all scenarios.

### 2.5. Spatial Changes and Centroid Shifts in Suitable Habitats for Broad-Leaved and Coniferous Species

**Figure 7:**
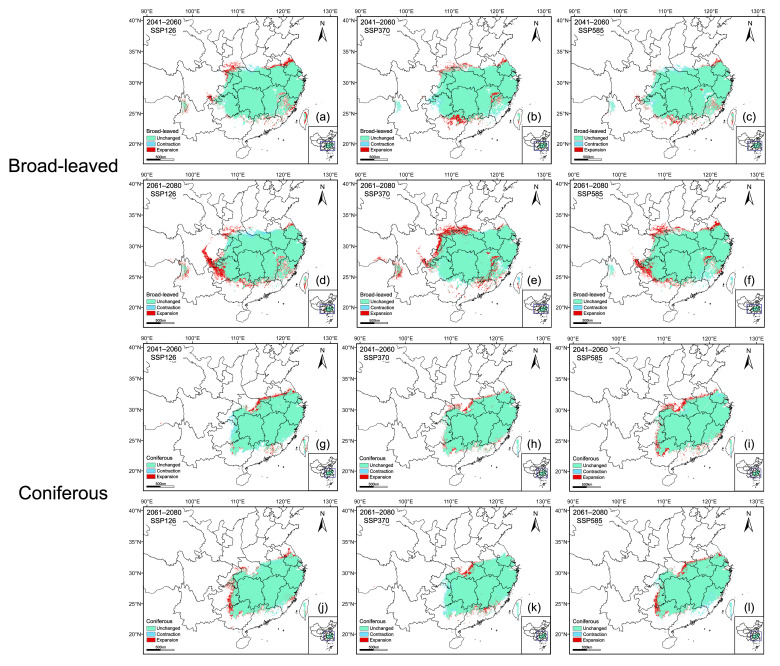
Spatial variation in potentially suitable habitats of broadleaved species (**a**–**f**) and coniferous species (**g**–**l**) under future climate scenarios.

To assess the potential impacts of future climate scenarios on rare and endangered broad-leaved and coniferous tree species, we compared their current and projected distributions to predict areas of habitat expansion, stability, and contraction (Figure 7). The results indicate that broad-leaved species generally exhibit a pronounced westward expansion, with their spread concentrated in the southwestern and northwestern portions of their current range, with only a little contraction in southern Henan (Figure 7a–f). In contrast, coniferous species are expected to mainly expand in the northern and western parts of their current distribution, with contraction primarily restricted to western Chongqing, western Guizhou, and southern Taiwan (Figure 7g–l).

**Figure 8:**
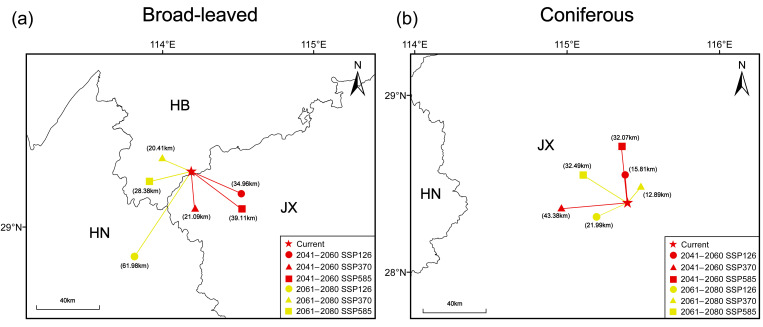
Changes in the centroids of suitable habitats for broad-leaved species (**a**) and coniferous species (**b**) under climate change scenarios. HB: Hubei province; HN: Hunan province; JX: Jiangxi province.

We further evaluated spatial shifts under future climate scenarios by calculating the centroid of suitable habitats for each species. The results reveal marked differences in centroid migration between broad-leaved and coniferous species. Broad-leaved species primarily shift toward lower latitudes and inland areas, with the longest and shortest migration distances occurring under SSP126 and SSP370 scenarios for 2061–2080, measuring 61.98 km and 20.41 km, respectively (Figure 8a). Conversely, coniferous species generally migrate towards higher latitudes, with the maximum and minimum migration distances observed under SSP370 for 2041–2060 and 2061–2080 being 43.38 km and 12.89 km, respectively (Figure 8b).

## 3. Discussion

### 3.1. Divergent Environmental Drivers for Broad-Leaved and Coniferous Species Distribution

Coniferous species and broad-leaved species could be expected to have different responses to climate change because they have dissimilar evolutionary histories, and distinct biological and ecological characteristics. Guo et al. suggested that temperature-related variables primarily limit coniferous species, while precipitation factors are more critical for broad-leaved taxa in China as a whole [2]. Our findings reveal differences in the primary environmental drivers shaping the potential habitats of the investigated broad-leaved and coniferous species in the subtropical regions of China. Thus, we determined that the distribution of broad-leaved species was predominantly governed by thermal factors (Bio11 and Bio2), whereas coniferous species exhibited a higher sensitivity to moisture availability (Bio17) (Table S2, Figure 3). This discrepancy is probably the result of differences in species’ ecological diversity, geographic range, and the specific physiological constraints of relict versus dominant species. In our study, we only selected species from subtropical forests, while the previous study included more species with broader geographic and ecological parameters.

For the five broad-leaved species studied, the mean temperature of the coldest quarter (Bio11) emerged as the most significant predictor (Table S2). This aligns with the ‘Refugia’ hypothesis for Tertiary relict plants in subtropical China [26]. In contrast to the widely distributed dominant broad-leaved species analyzed by Guo et al. (e.g., *Betula platyphylla* and *Ulmus pumila*) [2], which are often constrained by water availability in arid–semiarid transition zones of China, the endemic and endangered broad-leaved species examined in this study are largely restricted to humid subtropical evergreen broad-leaved forest regions. Under such conditions, water availability rarely constitutes a limiting factor; instead, the mean temperature of the coldest quarter (Bio11) acts as a key physiological filtering mechanism. Previous studies of the deciduous relict *Shaniodendron subaequale* have identified Bio11 as both a key factor for breaking seed dormancy and a critical indicator for safeguarding flower buds during the germination phase [27]. Since its seeds mature in late autumn and are prone to dehydration, stable winter temperatures are essential for maintaining seed viability and ensuring successful recruitment in specialized habitats such as gullies and slopes [15]. Furthermore, compared to widespread broad-leaved species, these endemic relict species often possess more specialized hydrothermal and reproductive requirements, making them vulnerable to frost damage or phenological transition failures under low winter temperatures [28,29]. The importance of the mean diurnal range (Bio2) further indicates that these species are more sensitive to environmental changes associated with temperature fluctuations (Table S2), a common characteristic of narrowly distributed endemic species occupying specialized ecological niches [30].

In contrast, our results indicate that the distribution of the endemic and endangered subtropical conifers (e.g., *Pinus dabeshanensis* and *Pseudotsuga gaussenii*) is primarily constrained by the precipitation of the driest quarter (Bio17) (Table S2, Figure S1). Although conifers generally exhibit greater drought tolerance than broad-leaved species due to their tracheid-based xylem structure and xeromorphic leaf traits [31], the coniferous species examined in this study are typically found on skeletal soils or rock outcrops in the Yangtze River basin, where annual precipitation is relatively high but seasonal water-holding capacity is low [32,33]. The occurrence of *P. dabeshanensis* on cliffs and mountain tops in the Dabie Mountains exposes the species to ‘physiological drought’ during dry periods. Although this species possesses an extensive root system capable of penetrating rock fissures, it largely depends on sustained atmospheric humidity and cloud or fog cover to reproduce [34]. During the driest quarter (Bio17), insufficient precipitation can lead to severe reductions in plant water potential, which may further exacerbate the already high rates of seed abortion and seedling mortality [35]. Consequently, Bio17 represents a critical limiting factor for these subtropical coniferous species. This pattern contrasts with that of widely distributed conifers (e.g., *Larix gmelinii*) studied by Guo et al., which extend into higher latitudes and elevations where very cold temperatures, rather than moisture availability, constitute the primary constraint on metabolic activity [2]. Our findings support the view that for the endemic and endangered conifers in the subtropics, niche conservatism associated with seasonal moisture stress is the principal cause of their fragmented distribution [26,31].

### 3.2. Differential Geographic Shifts Between Coniferous and Broad-Leaved Species Under Future Climate Conditions

Our results indicate that future climate change will induce distinct and contrasting geographical responses between rare and endangered coniferous and broad-leaved tree species in subtropical China. Although the core areas of both plant groups remain largely stable and will continue to be concentrated in eastern and central China, notable differences emerge in the direction of range expansion, contraction patterns, and centroid migration (Figure 5, Figure 6, Figure 7 and Figure 8), highlighting divergent ecological strategies and climatic sensitivities between these two functional groups.

Under future climate scenarios, broad-leaved species exhibit an overall tendency towards range expansion, particularly characterized by a westward and southward shift in their habitats. Expansion will be mainly concentrated along the southwestern and northwestern margins of the current distribution, whereas contraction will be limited and largely confined to southern Henan (Figure 7 and Figure 8). This pattern confirms that future climate warming is more likely to reshape marginal areas rather than substantially reduce core habitats [36]. Rising temperatures could alleviate low-temperature constraints in inland regions and prolong the growing season, and enhance thermal suitability, while concurrent increases in precipitation would favor the establishment and persistence of broad-leaved species [37]. Nevertheless, the effect of precipitation is seasonally heterogeneous. Although overall moisture availability may increase under future climate scenarios, precipitation during the driest month (Bio14) remains an important factor shaping habitat suitability at the distribution margins, as indicated by its high independent information content in the Jackknife analysis (Figure 3). This implies that seasonal water limitation may persist locally, particularly during the most drought-prone periods [38]. Even so, when considered together with improved thermal conditions, the net climatic effect is likely to be positive in many marginal areas, allowing former climatically constrained regions to transition towards higher suitability under future scenarios. Similar marginal expansion trends have been widely reported for subtropical broad-leaved taxa (e.g., *Quercus jenseniana*, *Quercus ciliaris*, and *Acer pubinerve*) [12,38]. This phenomenon is attributed to their physiological plasticity and broad climatic tolerances, which enables them to effectively track shifting climatic niches during increasing warmth [3,5,39].

In contrast, coniferous species exhibit a more complex response to future climate change that is strongly dependent on specific scenarios. Although an expansion is forecast for 2041–2060, this proliferation appears to be transient, followed by a pronounced contraction under certain circumstances, e.g., the SSP126 scenario during 2061–2080 and within highly suitable habitats under SSP370 (Figure 5). Our analysis of areas showing contraction of highly suitable habitats under the SSP370 scenario (2061–2080) indicates a marked decline in Bio17 values relative to current conditions, with projected conditions shifting away from the optimal range inferred from the species-specific response curves (Figures S2 and S3). These results suggest that moderate warming may initially relax thermal constraints and allow short-term range expansion, whereas continued climate change, together with increasing seasonal moisture limitation, may push environmental conditions beyond the physiological tolerance thresholds of subtropical coniferous species and ultimately lead to habitat contraction [40,41]. Spatially, expansion will mainly proceed towards the northern and western parts of the current distribution, indicating a tendency to track cooler climatic conditions, while contraction will occur in western Chongqing, western Guizhou, and southern Taiwan (Figure 6 and Figure 7). These regions are characterized by complex topography and pronounced climatic heterogeneity, where increasing temperatures combined with altered precipitation patterns could intensify heat stress and moisture imbalance, thereby preventing the survival of cryophilic coniferous taxa [42,43]. Similar patterns of contraction or instability in conifers under future warming scenarios have been widely reported in East Asia and other temperate regions (e.g., *Picea*, *Pseudotaxus chienii*, and *Torreya jackii*) [20], highlighting the general vulnerability of coniferous species to rising temperatures and increasing climatic variability compared with broad-leaved species [44,45].

Although detectable changes are likely at the margins of suitable habitats, the centroids of potential distributions for both coniferous and broad-leaved species are expected to remain relatively stable under future climate scenarios (Figure 7 and Figure 8). This limited centroid displacement indicates that projected climate change is unlikely to induce large-scale redistribution of these rare and endangered subtropical tree species in the short- to mid-term, with core areas continuing to function as climatically stable strongholds. Such stability suggests that future warming will primarily alter the spatial configuration and extent of marginal habitats rather than fundamentally shifting the geographic centers of species distributions, as previous research on subtropical tree species in China suggested [11,46].

### 3.3. Conservation Implications and Future Perspectives

Climate change, characterized by rising temperatures and altered precipitation patterns, poses an increasing challenge to the conservation of rare and endangered tree species in subtropical China. The results of this study indicate the critical necessity of implementing conservation strategies tailored to different functional groups within this region. We found a high degree of spatial overlap between current and future habitats, especially in the mountainous areas of Zhejiang, Jiangxi, Hunan, Hubei, and Anhui provinces (Figure 4, Figure 6 and Figure 7). These regions, such as the Dabie and Tianmu Mountains, will probably serve as climatically stable refugia, providing long-term buffering against regional climatic variability and extreme events [26,47]. Therefore, maintaining the integrity of these core habitats through strict in situ protection should be regarded as the primary management priority for both coniferous and broad-leaved species. Outside the core areas, conservation strategies should be differentiated according to ecological constraints. For broad-leaved species that exhibit inland and marginal expansion under warming conditions, the establishment and maintenance of habitat corridors facilitating westward and southward migration will enhance population persistence and adaptive capacity [3,5]. In contrast, subtropical conifers are more sensitive to seasonal moisture limitations, highlighting the importance of protecting habitats with stable hydrological conditions to support gradual range adjustments under future climate change [42,48]. We suggest that conservation planning for rare tree species in the middle and lower reaches of the Yangtze River should not rely on uniform protection measures, but should take the needs of the individual species into consideration.

This study employed an optimized MaxEnt model, integrating 19 climatic variables, 16 edaphic variables, and 3 topographic variables to predict the potential distribution of rare tree species in subtropical evergreen broad-leaved forests. While the model provides a robust framework for assessing these distributions (Figure 1 and Figure 2), several caveats regarding its predictive scope should be noted. First, non-climatic factors, such as interspecific competition, land-use change and anthropogenic disturbances, were not explicitly included in the model, which may lead to the overestimation of suitable habitats and centroid shifts [37,49]. Second, the coarse resolution of environmental layers may overlook fine-scale habitat heterogeneity, which is particularly critical for conifers with narrow distributions that are sensitive to seasonal moisture constraints [31]. Third, while our analysis yields reliable insights at the functional group level, we recognize that species with extensive geographic ranges may exhibit intraspecific variation in environmental adaptation. Local species might develop specific tolerances to regional climatic conditions through long-term adaptation or plasticity [20]. However, due to the inherent rarity of these endangered taxa and the restricted number of high-quality occurrence records, this study focused on the collective response of functional groups to ensure statistical stability and reliability. To address these limitations, future research should integrate high-resolution environmental variables alongside genomic data and experiments to further elucidate the nuances of local adaptation and niche differentiation within these lineages [50,51]. Furthermore, field validation in our projected habitats, particularly at range margins and potential expansion zones, is crucial for assessing the reliability of model outputs and guiding practical conservation interventions. Long-term monitoring programs that integrate species distribution modeling with field population surveys are of great significance for understanding the dynamic responses of broad-leaved and coniferous species during ongoing climate change [51]. Finally, scenario-based conservation planning should fully account for potential shifts in future climatic and land-use patterns to formulate adaptive strategies, thereby ensuring the long-term persistence of rare tree species under diverse and uncertain environmental conditions [52].

## 4. Materials and Methods

### 4.1. Compilation and Refinement of Occurrence Data

We selected nine representative endemic and endangered tree species in the subtropical evergreen broad-leaved forests of China, including five broad-leaved taxa *Shaniodendron subaequale*, *Litsea auriculata*, *Ulmus elongata*, *Calycanthus chinensis*, and *Sinojackia xylocarpa*, as well as four coniferous taxa *Torreya jackii*, *Pinus dabeshanensis*, *Pseudolarix amabilis*, and *Pseudotsuga gaussenii*. These occurrence records span a geographic range of approximately 109.48–122.39° E and 25.11–33.62° N, primarily encompassing key mountainous regions such as the Tianmu, Dabie, and Wuyi Mountains. Comprehensive records for the nine endemic and endangered plants were integrated from multiple authoritative sources: (1) our field investigations conducted in October 2024; (2) reliable biodiversity platforms and taxonomic references, including the ‘Chinese Virtual Herbarium’ (CVH, https://www.cvh.ac.cn/), ‘Chinese Field Herbarium’ (CFH, https://www.cfh.ac.cn/), the ‘Plant Photo Bank of China’ (PPBC, https://ppbc.iplant.cn/ ), the ‘Catalogue of Life China 2024 Annual Checklist’, and the ‘Flora of China’ (all accessed in November 2024); and (3) peer-reviewed academic literature.

**Figure 9:**
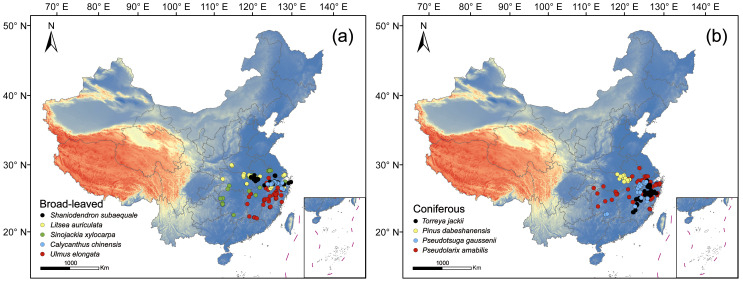
Spatial distribution of nine rare and endangered plants. (**a**) distribution of five broad-leaved species; (**b**) distribution of four coniferous species.

To ensure dataset integrity, we implemented a rigorous quality control protocol. To start with, doubtful records, cultivated material and duplicate entries were excluded. For specimens lacking precise georeferencing, coordinates were meticulously retrieved using Google Earth (http://www.google.cn, accessed on 1 November 2024) based on descriptions of the localities. To mitigate the risks of spatial autocorrelation and sampling bias, we employed a spatial thinning procedure, retaining only a single occurrence within each 1 × 1 km grid cell, consistent with the spatial resolution of the environmental predictors used in this study [12,53]. This refinement produced a robust spatial dataset for subsequent ecological niche modeling. Following these screening and thinning procedures, a final dataset comprising 189 valid records for broad-leaved species and 147 for the conifers was established for the MaxEnt 3.4 (American Museum of Natural History, New York, NY, USA) simulations (Figure 9, Table S1), which also provides the data source and distribution location for each occurrence record.

### 4.2. Environmental Data Acquisition and Multicollinearity Screening

To characterize the ecological requirements of the target species, we assembled a multi-dimensional suite of environmental predictors at a 30 arc-second (~1 km^2^) spatial resolution. (1) Climatic and Topographic Data: 19 bioclimatic variables and three topographic factors (elevation, slope, and aspect) were retrieved from the WorldClim (https://www.worldclim.org/, database accessed December 2025) for both current- and future scenarios. The future projections for 2041–2060 and 2061–2080 were based on the BCC-CSM2-MR model under three Shared Socioeconomic Pathways (SSP126, SSP370, and SSP585), representing optimistic, intermediate, and pessimistic climate trajectories, respectively [19,53,54]. This specific model was employed due to its proven efficacy in simulating the East Asian monsoon dynamics and temperature-precipitation variability across China [55,56]. Given the restricted distribution of our target species, utilizing a model validated for the Chinese climate ensures the geographic relevance and precision of our projections. (2) Edaphic Data: In addition to climatic factors, 16 fundamental soil indicators were sourced from the Harmonized World Soil Database (HWSD) (https://www.fao.org/, accessed December 2025) to account for the influence of substrate conditions on species distribution (Table S2).

To mitigate the risk of model overfitting and minimize the impacts of multicollinearity, a rigorous two-step screening procedure was executed [57,58]. Initially, we conducted a preliminary MaxEnt simulation and utilized ArcGIS 10.8 (Esri, Redlands, CA, USA) to extract environmental values at each location. Subsequently, we performed a statistical evaluation in R, employing Pearson correlation analysis (via the ‘cor’ function) and Variance Inflation Factor (VIF) calculations (using the ‘usdm’ package) [59]. Variables were prioritized for the final model based on their known biological significance as rare plants, with exclusion criteria set at an absolute correlation coefficient r ≥ 0.8 and a VIF ≥ 10 [12]. This refinement process ensured that the final subset of variables possessed high independent explanatory power for the distribution of both broad-leaved and coniferous species.

### 4.3. MaxEnt Model Optimization and Performance Validation

The predictive performance of the MaxEnt model is highly sensitive to the Regularization Multiplier (RM) and Feature Combinations (FCs). We utilized the ‘ENMeval’ package in R 3.1.2 (R Core Team, Vienna, Austria) to systematically optimize these parameters. We evaluated eight RM values (0.5 to 4.0, increment of 0.5) and six FC configurations (L, LQ, H, LQH, LQHP, and LQHPT), testing a total of 48 parameter permutations [60]. The optimal model configuration was identified by selecting the combination that minimized the corrected Akaike Information Criterion (AICc).

Final simulations were executed using MaxEnt 3.4 and ArcGIS 10.8. For each species, 75% of the occurrence data served as the training set, while the remaining 25% was reserved for testing, supplemented by 10,000 random background points. To ensure statistical stability, each simulation was replicated 10 times [19]. The Jackknife test of variable importance was conducted within each replicate model, and the results presented represent the mean response across all replicate runs, thereby reducing the influence of sampling variability on estimates of independent information value and model gain, with species-level results provided in the Supplementary Materials (Figure S1). Model accuracy was rigorously assessed using the Area Under the Curve (AUC) of the Receiver Operating Characteristic (ROC) curve. To verify the model’s generalization capability and detect potential overfitting, we reported and compared both Training AUC and Test AUC (calculated from the 25% validation subset, Table S3). Values exceeding 0.8 indicated high discriminative power and reliable predictive performance [12,19].

### 4.4. Spatial Classification and Distribution Dynamics Analysis

The continuous probability outputs from MaxEnt were reclassified within ArcGIS 10.8 into four distinct suitability tiers. To ensure biological rationality, the baseline threshold for presence was determined based on the 10 percentile presence threshold (10P) obtained from model performance metrics (Table S3). Specifically, the average 10P value (~0.11 for broad-leaved and ~0.13 for coniferous species) was utilized to define the boundary between unsuitable and suitable habitats. For the areas identified as suitable (*p* ≥ 10P), the natural breaks method was further applied to objectively partition the remaining probability range into three refined tiers to reflect varying degrees of habitat quality: unsuitable (*p* < 10P), barely suitable (10P ≤ *p* < ~0.3), moderately suitable (~0.3 ≤ *p* < ~0.5), and highly suitable (*p* ≥ ~0.5) [11].

To recognize the spatial shifts induced by climate change, we utilized the SDMtools 2.5 plugin. Probability maps were first converted into binary (presence/absence) rasters using the 10 percentile presence threshold as the cutoff. By comparing future projections against current distributions, we mapped and quantified ‘unchanged’, ‘expansion’, and ‘contraction’ habitat regions [12,55]. Furthermore, we calculated the spatial centroid for each period to determine the direction and magnitude of the distributional shift, thereby elucidating the evolutionary trajectory of these rare species under various climate change trajectories.

## 5. Conclusions

This study employed an optimized MaxEnt model to systematically assess the distributional dynamics of nine endemic and endangered coniferous and broad-leaved tree species in subtropical evergreen broad-leaved forests of China under current and future climate scenarios. We found that the distribution of rare subtropical broad-leaved species is primarily controlled by temperature-related factors, whereas rare conifers show a stronger dependence on water availability, with the precipitation of the driest quarter (Bio17) being the key limiting factor. Under future climate scenarios, broad-leaved species generally show a tendency to expand westward and towards lower latitudes, while coniferous species display more complex and fluctuating responses, predominantly exhibiting a northward shift. Highly suitable areas for both functional groups remain relatively stable in multiple future climate scenarios, with core habitats located in mountainous and hilly regions of provinces such as Zhejiang, Jiangxi, Anhui, Hunan, and Hubei (e.g., Tianmu Mountains, Dabie Mountains), which will probably serve as refugia for rare subtropical tree species. This study demonstrates that the responses of endemic and endangered subtropical tree species to climate change are not only constrained by regional climatic conditions, but are also significantly influenced by functional group attributes, highlighting the need to develop spatially targeted strategies which take the ecological constraints of the different functional groups into consideration.

## Data Availability

The original contributions presented in this study are included in the article/Supplementary Material. Further inquiries can be directed to the corresponding author.

## Supplementary Materials

The following supporting information can be downloaded at https://www.mdpi.com/article/10.3390/plants15030515/s1, Table S1: Distribution data of coniferous and broad-leaved tree species; Table S2: Environmental variables used for the construction of the Maxent model; Table S3: Accuracy metrics and presence thresholds for forest SDMs; Figure S1: Jackknife test results for environmental variables influencing the potential distributions of the nine studied tree species; Figure S2: Spatial patterns of contraction in highly suitable habitats for coniferous species under the SSP370 scenario (2061-2080); Figure S3: Response curve of coniferous species to precipitation of the driest quarter (Bio17), with current and SSP370 (2061-2080) conditions in contracted areas indicated. Note: Dashed lines indicate mean Bio17 values extracted from contracted high-suitability areas under current and SSP370 (2061-2080) conditions.
