# Supplementary material for: Differential Performance of Distribution Shifts Between Endangered Coniferous and Broad-Leaved Tree Species in Subtropical China Under Climate Change
Source: Plants (Basel). 2026 Feb 6;15(3):515. doi: 10.3390/plants15030515 (PMC12899582; doi:10.3390/plants15030515)
Supplement: Supplementary file 1 [file plants-15-00515-s001.zip › Supplementary Materials.pdf]

Differential performance of distribution shifts between endangered coniferous and broad-leaved tree species in subtropical China under climate change

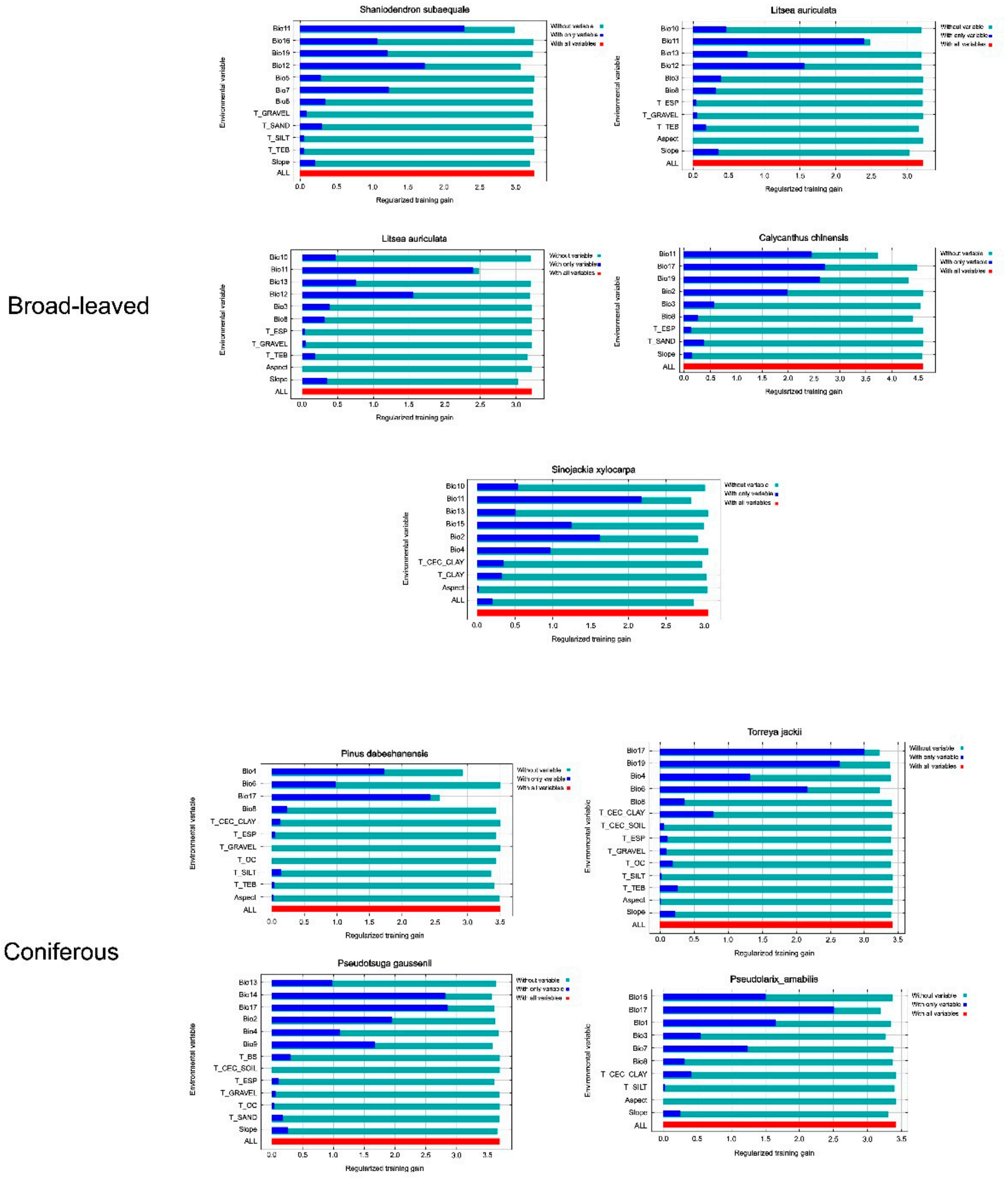

Figure S1. Jackknife test results for environmental variables influencing the potential distributions of the nine studied tree species.

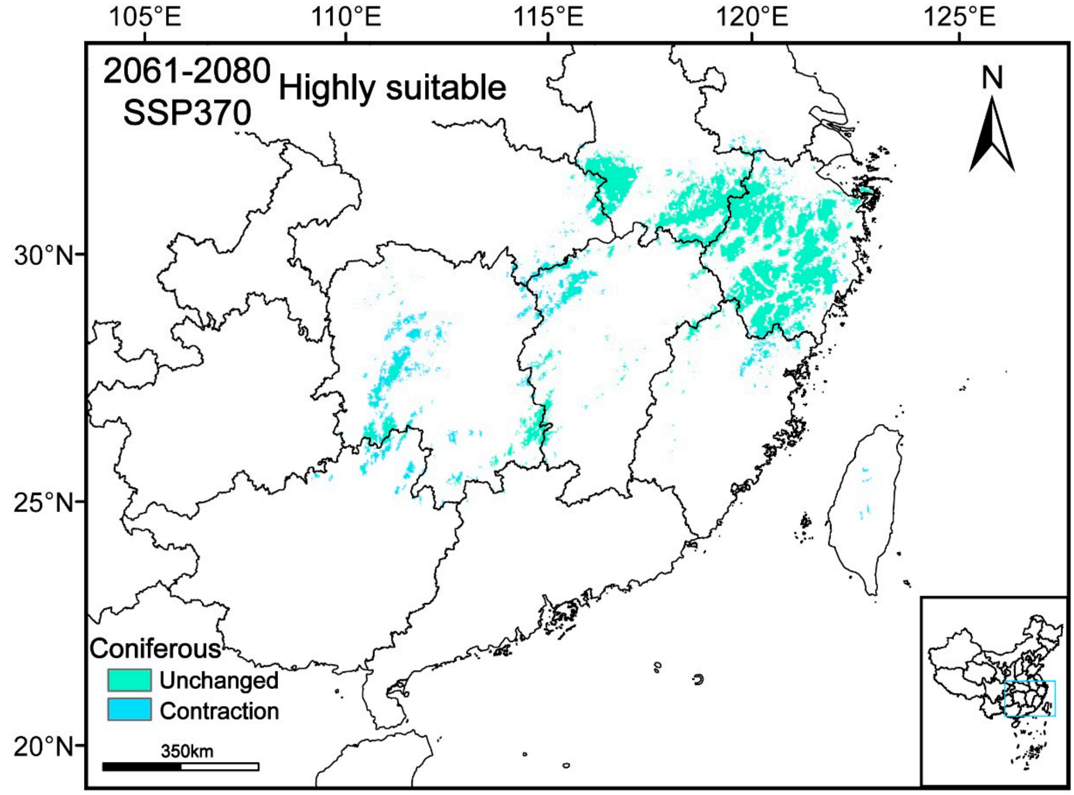

Figure S2. Spatial patterns of contraction in highly suitable habitats for coniferous species under the SSP370 scenario (2061-2080)

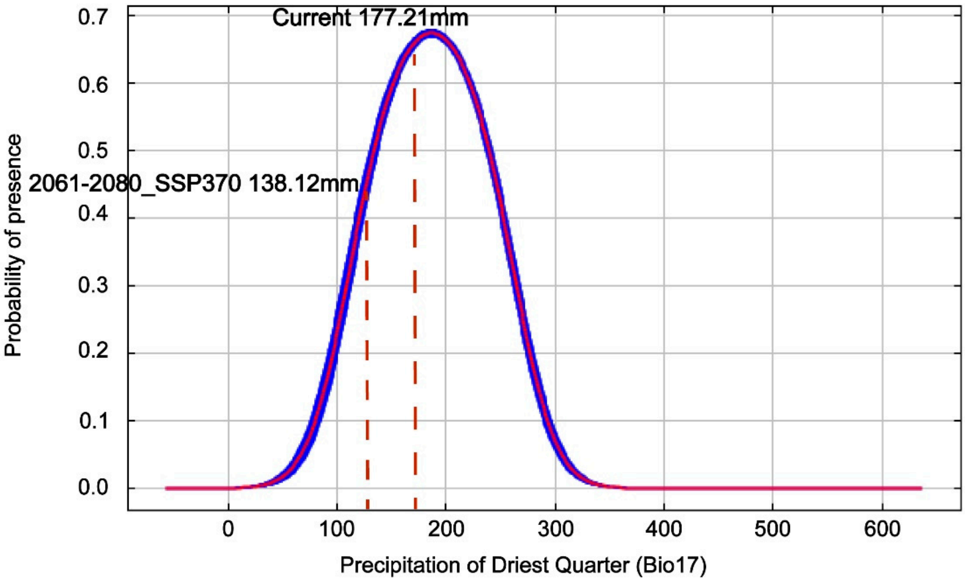

Figure S3. Response curve of coniferous species to precipitation of the driest quarter (Bio17), with current and SSP370 (2061-2080) conditions in contracted areas indicated. Note: Dashed lines indicate mean Bio17 values extracted from contracted high-suitability areas under current and SSP370 (2061-2080) conditions.
